# Prolactin Response to a Submaximal Dose of Ghrelin in Different Phases of the Normal Menstrual Cycle

**DOI:** 10.3390/medicina60122039

**Published:** 2024-12-11

**Authors:** Christina I. Messini, George Anifandis, Panagiotis Georgoulias, Konstantinos Dafopoulos, George Sveronis, Alexandros Daponte, Ioannis E. Messinis

**Affiliations:** 1 Department of Obstetrics and Gynaecology, Faculty of Medicine, School of Health Sciences, University of Thessaly, 41500 Larissa, Greece; messini@uth.gr (C.I.M.); ganif@uth.gr (G.A.); kdafop@uth.gr (K.D.); sveron.giorgos@yahoo.gr (G.S.); daponte@uth.gr (A.D.); 2 Department of Nuclear Medicine, Faculty of Medicine, School of Health Sciences, University of Thessaly, 41500 Larissa, Greece; georgoulias@hotmail.com

**Keywords:** ghrelin, prolactin, growth hormone, normal menstrual cycle

## Abstract

***Background and Objectives*:** A similar secretory pattern of prolactin (PRL) and growth hormone (GH) during the menstrual cycle has been reported in response to a high dose of ghrelin in adult healthy women. The present study aimed to assess the pattern of PRL and GH secretions in response to a submaximal dose of ghrelin during different menstrual phases in adult healthy women. ***Materials and Methods*:** Eight female subjects with normal cyclicity were enrolled. These subjects were either in the early follicular (EF), late follicular (LF), or mid-luteal (ML) phase of their cycles. Each subject received an IV dose of normal saline (2 mL each time) during the first cycle after enrollment, followed by an IV dose of ghrelin (0.30 μg/kg bw) in the second cycle. The blood samples were collected before and after the IV dosage at −15, 0, 15, 30, 45, 60, 75, 90 and 120 min, where 0 min denotes the time of IV dosage. ***Results*:** All the enrolled subjects experienced ovulatory cycles as assessed by increased serum progesterone levels. Serum estradiol levels were significantly higher in the LF than in the EF (*p* < 0.001) and ML phases (*p* < 0.01); these levels were also significantly higher in the ML than in the EF phase (*p* < 0.01). The administration of saline did not affect serum GH or PRL levels. Following the administration of ghrelin, plasma ghrelin levels and serum GH levels increased significantly (*p* < 0.001). The response amplitude of GH was similar in the three stages of cycle 2. In contrast to GH, the ghrelin injection induced a significant increase in serum PRL levels only in the LF phase (*p* < 0.05). ***Conclusions*:** These results show, for the first time, a different pattern of PRL and GH in response to a submaximal dose of ghrelin during the normal menstrual cycle. It is suggested that the ghrelin threshold for pituitary lactotrophs is higher than for somatotrophs and that, unlike GH, ghrelin-stimulated PRL secretion can be influenced by ovarian steroids.

## 1. Introduction

Ghrelin is an orexigenic 28-amino-acid peptide primarily secreted by the mucosa of the stomach [1,2]. This protein is considered the endogenous ligand of the growth hormone (GH) secretagogue receptor type 1a (GHS-R1a) [1,3,4]. It has been shown that ghrelin administration at a high dose of 1 μg/kg stimulates the secretion of GH in women during the normal menstrual cycle with no difference in the response between the follicular and the luteal phase, suggesting that this effect of ghrelin is not influenced by ovarian steroids [5]. Although the 1 μg/kg dose produces strong pituitary stimulation regarding GH secretion, smaller doses, characterized as submaximal stimulatory amounts of ghrelin [6], have been used on a limited basis in research studies. For example, the effect of 0.15 μg/kg and 0.30 μg/kg doses on GH secretion has only been tested in the follicular phase of the cycle [7], while their effect during the luteal phase is not known.

Apart from GH, ghrelin also stimulates the secretion of prolactin (PRL) in humans [8]. The action of ghrelin on PRL secretion appears to be mediated via the receptor GHS-R1a, as in GHS-R-/- mice there was a reduction in PRL mRNA expression [9]. Regarding the normal menstrual cycle, a significant increase in PRL secretion in response to a high ghrelin dose (1 μg/kg) has been demonstrated that did not differ significantly between the early and the late follicular phases, as well as the mid-luteal phase, suggesting that this effect of ghrelin is independent of the levels of estrogen and progesterone [5]. However, the effect of lower doses of ghrelin on PRL secretion has not been investigated. This would be of interest if we consider previous data regarding gonadotropin secretion, showing that a high dose of ghrelin (1 μg/kg) did not alter FSH and LH levels throughout the menstrual cycle [10], while two submaximal doses of this protein (0.15 and 0.30 μg/kg) suppressed gonadotropin levels during the follicular phase [11]. Whether a differential pituitary response to varying doses of ghrelin also applies to PRL secretion remains to be examined.

The present study was undertaken to investigate the hypothesis that a submaximal dose of ghrelin induces a different pattern of PRL response in the different phases of the normal menstrual cycle.

## 2. Materials and Methods

### 2.1. Patients

Eight women aged 20–32 years (mean 24.9 years) with normal menstruation cycles volunteered for the study. All women were healthy, with a normal body mass index (BMI), and had not received any hormonal or any other medical treatment during the last 6 months before entering the study. Ovulation was confirmed in all women with serum progesterone measurement on day 21 of the cycle before entering the study.

### 2.2. Experiments

Each woman was investigated across two consecutive normal menstrual cycles. In cycle 1 (control), the women received an acute IV injection of normal saline (2 mL) 3 times during the cycle, i.e., in the early follicular phase (day 3 of the cycle), in the late follicular phase (when the dominant follicle was 16–17 mm in mean diameter assessed by ultrasound), and in the mid-luteal phase (7 days following ovulation assessed by ultrasound). In cycle 2, the women received an acute IV injection of ghrelin (Ghrelin, C-S-142, Clinalfa, Merck Biosciences AG, Läufelfingen, Switzerland) at a dose of 0.30 μg/kg in the three stages of the cycle, as described in cycle 1. The total number of experimental procedures carried out on the 8 women was 48. All experimental procedures took place in the morning between 10.00 and 12.00 h after overnight fasting of the women. Before each experimental procedure, the women were placed in the supine position to relax for 15 min. Then, an intravenous catheter was inserted into a forearm vein, and the first blood sample was taken 15 min later (time −15 min). The second blood sample was taken 15 min after the first and immediately before the injection of the saline or ghrelin (time 0 min). Further blood samples were obtained from all women at 15, 30, 45, 60, 75, 90 and 120 min. After each sampling, the catheter was heparinized. Ghrelin, GH and PRL levels were measured in all blood samples. Basal levels of FSH, LH, estradiol and progesterone were also measured in the samples, but at −15 min of all experiments. Blood samples were centrifuged at 1000× *g* for 15 min, and the plasma and serum were stored at −20 °C until assayed.

### 2.3. Hormone Assays

Total ghrelin levels were measured in plasma by using a radioimmunoassay (KIPMR90, BioSource Europe S.A., Nivelles, Belgium). The results are expressed as pg/mL. GH, FSH, LH and PRL were measured in serum via an immunoradiometric assay (hGH-IRMA, FSH-IRMA, LHsp-IRMA and PRL-IRMA, respectively, BioSource Europe S.A.); the results are expressed as ng/mL, mIU/mL, mIU/mL and ng/mL, respectively. Measurement of estradiol and progesterone in serum was performed using a radioimmunoassay (E2-RIA-CT and PROG-RIA-CT, respectively, BioSource Europe S.A.) and the results are expressed as pg/mL and ng/mL, respectively. The lower limits of detection for ghrelin, GH, FSH, LH, PRL, estradiol and progesterone were 40 pg/mL, 0.07 ng/mL, 0.1 mIU/mL, 0.2 mIU/mL, 0.35 ng/mL, 2 pg/mL and 0.05 ng/mL, respectively, while the inter- and intra-assay coefficients of variation were 7.3 and 5.0%, 6.7 and 0.7%, 2.4 and 1.1%, 3.4 and 1.4%, 4.5 and 3.3%, 6.2 and 4.9%, and 6.5 and 3.3%, respectively.

### 2.4. Statistical Analysis

All hormone values showed a normal distribution (one-sample Kolmogorov–Smirnov test). Statistical evaluation was performed using repeated measures one-way analysis of variance (ANOVA) followed by Bonferoni post hoc testing. The α level of 0.05 was considered to be significance. All values are expressed as mean ± SEM. The statistical package NCSS 2001 (Number Cruncher Statistical Systems, Kaysville, UT, USA) was used.

## 3. Results

### 3.1. Basal Hormonal Values

Basal hormonal values of the women across the two cycles (time −15 min) are shown in Table 1. Serum LH values were higher in the late than in the early follicular phase due to the LH surge, but the difference was not significant due to the large standard deviation, suggesting that the LH surge did not start on the same day in all women. Serum progesterone levels in the mid-luteal phase increased in all women and the mean value was significantly higher than in the early and the late follicular phases (*p* < 0.001), confirming ovulation in both cycles. Serum estradiol concentrations were significantly higher in the late follicular than in the early follicular phase (*p* < 0.001) and the mid-luteal phase in both cycles (*p* < 0.01). Also, serum estradiol levels in the mid-luteal phase were significantly higher than in the early follicular phase in both cycles (*p* < 0.01). In each cycle, basal serum FSH, PRL and GH levels did not differ significantly between the three stages. Between the two cycles, there were no significant differences in the corresponding baseline values of the hormones in the three stages studied.

### 3.2. Effect of Saline Administration on Plasma Ghrelin and Serum GH and PRL Levels

**Figure 1:**
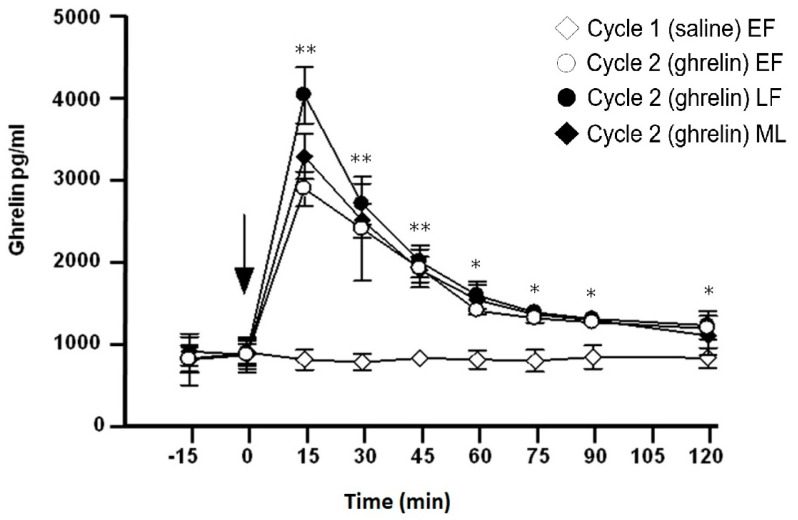
Plasma ghrelin values (pg/mL) before and after an acute IV injection (arrow) of normal saline (2 mL) to 8 healthy women during the (◊) early follicular phase (EF) of cycle 1 (control) as well as before and after an acute IV injection (arrow) of ghrelin (0.30 μg/kg) to the same women during the (○) EF, (●) late follicular (LF) and (♦) mid-luteal phase (ML) of cycle 2. ** *p* < 0.01, * *p* < 0.05 (difference from cycle 1).

**Figure 2:**
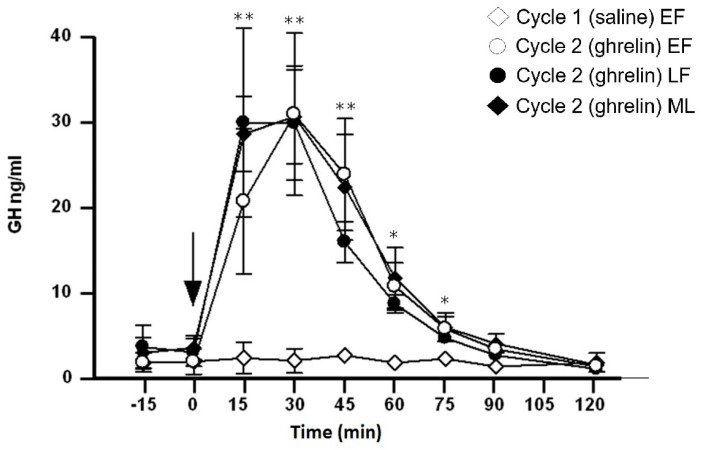
Serum growth hormone (GH) values (ng/mL) before and after an acute IV injection (arrow) of normal saline (2 mL) to 8 healthy women during the (◊) early follicular phase (EF) of cycle 1 (control) as well as before and after an acute IV injection (arrow) of ghrelin (0.30 μg/kg) to the same women during the (○) EF, (●) late follicular (LF) and (♦) mid-luteal phase (ML) of cycle 2. ** *p* < 0.01, * *p* < 0.05 (difference from cycle 1).

**Figure 3:**
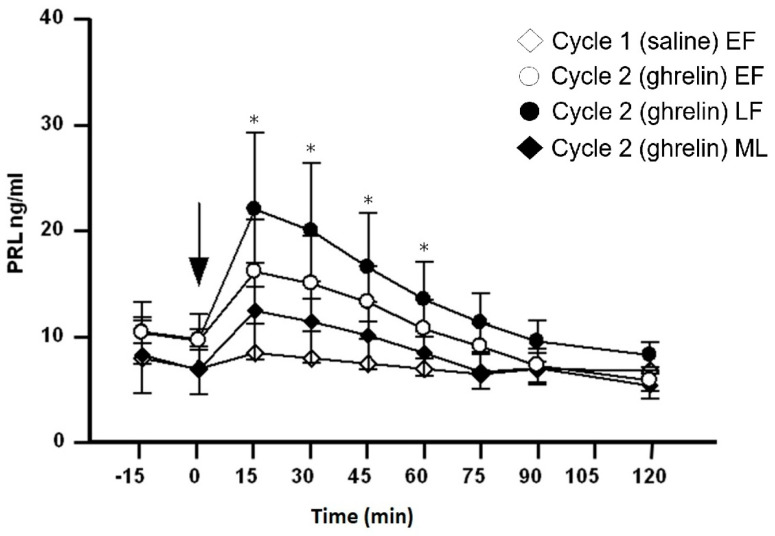
Serum prolactin (PRL) values (ng/mL) before and after an acute IV injection (arrow) of normal saline (2 mL) to 8 healthy women during the (◊) early follicular phase (EF) of cycle 1 (control) as well as before and after an acute IV injection (arrow) of ghrelin (0.30 μg/kg) to the same women during the (○) (EF), (●) late follicular (LF) and (♦) mid-luteal phase (MF) of cycle 2. * *p* < 0.05 (difference from cycle 1).

Basal values of ghrelin before the administration of saline or ghrelin were similar across the two cycles (−15 and 0 min). Plasma ghrelin values did not change after saline injection (Figure 1). After the administration of saline, no changes were observed in the studied hormones (GH, PRL) in all three stages of cycle 1. However, for reasons of simplification, only the values in the early follicular phase for cycle 1 are presented in Figure 1, Figure 2 and Figure 3.

### 3.3. Effect of Ghrelin Administration on Plasma Ghrelin Levels (Figure 1)

Administration of ghrelin resulted in a significant increase in plasma levels of this hormone across the three stages of cycle 2, with no significant difference between them. Peak values were seen at 15 min in all three stages of cycle 2. The difference at 15 min was significant from the basal value (time 0 min) (*p* < 0.001), and ghrelin values remained significantly higher than in cycle 1 from 15 to 120 min (*p* < 0.001).

### 3.4. Effect of Ghrelin Administration on Serum GH Levels (Figure 2)

Basal values of GH before the administration of saline or ghrelin were similar in the two cycles (−15 and 0 min). The administration of ghrelin significantly stimulated the secretion of GH; this pattern was similar across the three stages of cycle 2 with no significant differences in the GH levels between them. Maximum values were seen at 30 min (*p* < 0.001; difference from basal value at 0 min). Serum GH levels were significantly higher in cycle 2 than in cycle 1 from 15 to 75 min (*p* < 0.001).

### 3.5. Effect of Ghrelin Administration on Serum PRL Levels (Figure 3)

There was no significant difference in basal serum PRL values before the administration of saline or ghrelin between the two cycles (−15 and 0 min). In cycle 2, ghrelin administration resulted in a significant increase in serum PRL levels at the point of 15 min when compared to the point of 0 min, but only in the late follicular phase (22.5 ± 6.00 vs. 9.7 ± 0.95 ng/mL, *p* < 0.05). At the same time point (15 min), serum PRL levels in the late follicular phase of cycle 2 were significantly higher than in cycle 1 (8.2 ± 1.16 ng/mL, *p* < 0.05). Moreover, at 30 min (19.95 ± 5.5 ng/mL), 45 min (16.7 ± 3.70 ng/mL) and 60 min (13.5 ± 2.85 ng/mL) in the late follicular phase of cycle 2, serum PRL levels were significantly higher than in cycle 1 (7.85 ± 1.14, 7.0 ± 1.16 and 6.85 ± 1.15 ng/mL, respectively, *p* < 0.05). No other significant differences were observed at the other time points between the PRL levels of cycles 1 and 2.

## 4. Discussion

The present study shows for the first time that a submaximal dose of ghrelin administered to healthy women significantly stimulated the secretion of PRL during the late follicular phase of the normal menstrual cycle. The increase in PRL secretion was the result of ghrelin administration, which is evident first from the coincidentally marked increase in plasma ghrelin levels and secondly from the lack of PRL increase in the saline (control) group. The present results reject the null hypothesis of no difference in the response pattern and prove the alternative hypothesis. In fact, as opposed to the late follicular phase, the increase in PRL levels after the injection of ghrelin in the early follicular and the mid-luteal phases of the cycle was not significant. This pattern of pituitary responsiveness to a submaximal dose of ghrelin is distinct from that seen previously after the injection of a high dose of this protein (1 μg/kg), which caused a significant increase in PRL secretion in all three phases of the cycle with no significant difference between them [5]. A dissimilar pattern of pituitary response to ghrelin has also previously been observed regarding FSH and LH secretion in the follicular phase that was suppressed by two submaximal doses, but not the aforementioned high dose of ghrelin [10,11].

The reason for the different pattern of PRL response to the submaximal dose of ghrelin in the three phases of the cycle is not clear a priori. One important insight is that the large fluctuations of ovarian hormones during the menstrual cycle with serum estradiol levels are highest during the late follicular phase. It has been demonstrated that this steroid, whether endogenous or exogenous, can be an independent stimulus for PRL secretion in women [12]. Also, the amplifying effect of estradiol on ghrelin-induced PRL secretion has been shown in estrogen-deprived postmenopausal women [13]. It is, therefore, possible that the high serum estradiol levels in the late follicular phase sensitized the pituitary lactotrophs to the submaximal dose of ghrelin, resulting in the significant increase in PRL release, whereas in the early follicular phase the low amount of circulating estradiol was apparently insufficient for such action.

Regarding the mid-luteal phase, the failure of ghrelin to cause a significant increase in PRL levels could be due to the fact that at that stage of the cycle, serum estradiol levels, although significantly higher than in the early follicular phase, were not as high as those in the late follicular phase. Nevertheless, a more likely explanation is that the high progesterone levels in the luteal phase counteracted a potential sensitizing effect of estradiol on the pituitary PRL response to ghrelin. This is consistent, on one hand, with the general assumption that progesterone is the hormone that antagonizes the action of estrogen in various tissues [14,15] and, on the other hand, with previous findings in rats demonstrating that progesterone suppressed estrogen-stimulated PRL secretion in vitro [16].

If progesterone is the key player, it appears from the present data that the estrogen-neutralizing action of progesterone has an impact on the effect of only small doses of ghrelin, since higher doses overcome the action of this steroid thus achieving a similar range of response in the three stages of the cycle [5]. Another interpretation of the present data could be that the ghrelin threshold for PRL secretion is quite high, so that either higher amounts of this protein are required to exceed it, or, in the absence of high levels of progesterone, high estrogen levels render the low dose of ghrelin effective. Further research is needed to investigate this matter further.

The stimulating effect of ghrelin on GH secretion seen in the present study has also been shown previously with both a high dose and two submaximal doses of ghrelin [5,7]. Nevertheless, the experiments with submaximal doses of ghrelin were previously performed only in the follicular phase of the cycle, while investigation of the effect of a submaximal dose during the luteal phase was performed for the first time in the present study. Interestingly, unlike the great variability in PRL secretion, the GH response to ghrelin in the present study was consistent with a similar magnitude across the three stages of the menstrual cycle, suggesting that the GH secretion was not affected by ovarian steroids, just as with a high dose of ghrelin [5]. Nevertheless, the possibility that estrogen may affect ghrelin-induced GH secretion under certain conditions cannot be excluded according to previous data showing that the short-term administration of estrogen to postmenopausal women increased the stimulatory effect of a relatively low dose of ghrelin on pulsatile GH secretion [6] or augmented hypothalamo-pituitary sensitivity to ghrelin [17]. Furthermore, the potent estrogen receptor antagonist, fulvestrant, amplified fasting and ghrelin-stimulated GH secretion in postmenopausal women who were not treated with estrogen [18]. This topic, therefore, needs further investigation.

A limitation of the present study is the small number of women. Nevertheless, each woman was investigated across two menstrual cycles and thus served as her own control. In addition, the total number of experimental procedures in the eight women was 48, and from each woman a total of 54 blood samples were taken across the two cycles. It is clear that for such research interventions, it is difficult to recruit a large number of volunteers, and this may not be necessary if we consider the clear design (placebo controlled) and the stability and consistency of the GH results across the three stages of the cycle. The wide variety of PRL response to ghrelin compared to GH is probably related to the ghrelin threshold, which seems to be higher for pituitary lactotrophs than for somatotrophs, the latter responding to a submaximal dose of ghrelin without the interaction of ovarian steroids. This is also supported by previous data showing a stable and consistent depiction of GH values after the administration of an even lower dose of ghrelin (0.15 μg/kg) from that in the present study (0.30 μg/kg) [7]. Finally, it should be noted that the present results were derived from experimental procedures with a pharmacological approach; therefore, the extent to which they may reflect physiological hormonal interactions needs to be further investigated. Consequently, the clinical significance of the present data is difficult to assess at present.

## 5. Conclusions

The present study demonstrates, for the first time, that a submaximal dose of ghrelin given to healthy women via injection during the menstrual cycle induced a significant increase in serum PRL levels. However, in contrast to the consistent response of GH to the same dose of ghrelin, a different pattern of PRL response was found, indicating that the ghrelin threshold appears to be higher for pituitary lactotrophs than for somatotrophs. It is suggested that under the present experimental conditions, unlike GH, ghrelin-stimulated PRL secretion during the normal menstrual cycle can be influenced by ovarian steroids. It is evident that further research is required.

## Figures and Tables

**Table 1:** Basal serum hormone levels in the blood sample taken from 8 healthy women at −15 min of the three experiments performed during the early follicular (EF), late follicular (LF) and mid-luteal phase (ML) of cycle 1 (normal saline—control) and cycle 2 (ghrelin).

		Cycle 1			Cycle 2	
	EF	LF	ML	EF	LF	ML
FSH (mIU/mL)	4.8 ± 0.5	4.0 ± 0.7	3.1 ± 0.4	4.5 ± 0.5	4.3 ± 0.8	3.3 ± 0.6
LH (mIU/mL)	3.7 ± 0.4	16.3 ± 8.5	2.8 ± 0.9	4.1 ± 1.0	20.9 ± 10.1	2.9 ± 0.2
E2 (pg/mL)	43 ± 10.3	268 ± 32.7 ^a,b^	124 ± 24.6 ^c^	54 ± 13.2	363 ± 43.2 ^a,b^	169 ± 41.4 ^c^
P4 (ng/mL)	1.9 ± 0.7	2.6 ± 0.8	18.3 ± 2.1 ^a^	2.4 ± 0.8	3.1 ± 0.7	20.1 ± 2.9 ^a^
PRL (ng/mL)	8.1 ± 2.9	7.5 ± 1.1	10.1 ± 2.3	10.4 ± 2.9	10.5 ± 1.1	8.3 ± 3.6
GH (ng/mL)	1.6 ± 0.8	2.5 ± 0.8	2.1 ± 0.9	1.8 ± 1.0	3.5 ± 2.3	2.9 ± 1.7

E2: estradiol; P4: progesterone; PRL: prolactin; GH: growth hormone. For E2 (both cycles): ^a^ shows the difference between the LF and EF phase (*p* < 0.001), ^b^ shows the difference between the LF and ML phase (*p* < 0.01) and ^c^ shows the difference between the ML and EF phase (*p* < 0.01). For P4 (both cycles): ^a^ shows the difference between the ML and the other two phases (*p* < 0.001).

## Data Availability

Access to the data supporting the findings of this study may be provided by the corresponding author, [I.E.M.], upon reasonable request.
